# The level of serum albumin is associated with renal prognosis and renal function decline in patients with chronic kidney disease

**DOI:** 10.1186/s12882-023-03110-8

**Published:** 2023-03-15

**Authors:** Tong Cheng, Xiaoyu Wang, Yong Han, Jianbing Hao, Haofei Hu, Lirong Hao

**Affiliations:** 1 Department of Nephrology, Southern University of Science and Technology Hospital, No. 6019 Liuxian Street, Xili Avenue, Nanshan District, Shenzhen, Guangdong Province 518000 China; 2 Department of Nephrology, Hechi People’s Hospital, Hechi, Guangxi Zhuang Autonomous Region 547000 China; 3 Department of Emergency, Shenzhen Second People’s Hospital, Shenzhen, Guangdong Province 518000 China; 4 Department of Nephrology, Shenzhen Second People’s Hospital, No. 3002 Sungang Road, Futian District, Shenzhen, Guangdong Province 518000 China

**Keywords:** Chronic kidney disease, Serum albumin, Renal prognosis, Renal function decline, Cox proportional hazards regression model, Linear regression model

## Abstract

**Objective:**

The study’s purpose is to explore the link of serum albumin on renal progression in patients with chronic kidney disease (CKD).

**Methods:**

This study was a secondary analysis of a prospective cohort study in which a total of 954 participants were non-selectively and consecutively collected from the research of CKD-ROUTE in Japan between November 2010 and December 2011. We evaluated the association between baseline ALB and renal prognosis (initiation of dialysis or 50% decline in eGFR from baseline) and renal function decline (annual eGFR decline) using the Cox proportional-hazards and linear regression models, respectively. We performed a number of sensitivity analyses to ensure the validity of the results. In addition, we performed subgroup analyses.

**Results:**

The included patients had a mean age of (66.86 ± 13.41) years, and 522 (69.23%) were male. The mean baseline ALB and eGFR were (3.89 ± 0.59) g/dL and (33.43 ± 17.97) ml/min/1.73 m^2^. The annual decline in eGFR was 2.65 mL/min/1.73 m^2^/year. 218 (28.9%) individuals experienced renal prognosis during a median follow-up period of 36.0 months. The baseline ALB was inversely linked with renal prognosis (HR = 0.61, 95%CI: 0.45, 0.81) and renal function decline (β = -1.41, 95%CI: -2.11, -0.72) after controlling for covariates. The renal prognosis and ALB had a non-linear connection, with ALB’s inflection point occurring at 4.3 g/dL. Effect sizes (HR) were 0.42 (0.32, 0.56) and 6.11 (0.98, 38.22) on the left and right sides of the inflection point, respectively. There was also a non-linear relationship between ALB and renal function decline, and the inflection point of ALB was 4.1 g/dL. The effect sizes(β) on the left and right sides of the inflection point were -2.79(-3.62, -1.96) and 0.02 (-1.97, 1.84), respectively.

**Conclusion:**

This study shows a negative and non-linear association between ALB and renal function decline as well as renal prognosis in Japanese CKD patients. When ALB is lower than 4.1 g/dL, ALB decline was closely related to poor renal prognosis and renal function decline. From a therapeutic point of view, reducing the decline in ALB makes sense for delaying CKD progression.

**Supplementary Information:**

The online version contains supplementary material available at 10.1186/s12882-023-03110-8.

## Background

Globally, chronic kidney disease (CKD), which can progress to end-stage renal disease (ESRD), has emerged as a severe health concern [1]. It is associated with increased mortality and cardiovascular morbidity risk, resulting in a subsequent heavy social and economic burden [2–4]. In 2017, an estimated 700 million people were living with chronic kidney disease (CKD) and 1.2 million deaths worldwide [5]. CKD affects 13.3 million people, or about 13% of Japan’s adult population [6]. In addition, a rapid decline in renal function was also significantly associated with higher mortality and higher cardiovascular morbidity, independent of baseline renal function levels [7, 8]. A recent study suggested that a decline in estimated glomerular filtration rate (eGFR) of more than 30% over 2 years may indicate significant progression of CKD. The rate of eGFR decline may also provide additional prognostic information compared with conventional mortality risk factors in the CKD groups [9]. Studies in recent years have evaluated eGFR slope-based methods, using multiple measurements of eGFR to explore the associations between annual changes in eGFR and subsequent ESRD hazards [10]. Therefore, analyzing possible risk factors leading to renal damage and deterioration has become essential to preventing and treating renal disease.

ALB is the most abundant protein in human plasma, accounting for roughly half of the total protein content of the serum. It is a commonly used clinical biomarker. Even minor declines in blood albumin levels are substantially related to cardiovascular disease, heart failure, and mortality in sensitive populations, such as HIV-infected and elderly adults, according to previous research [11–13]. A recent US study revealed that decreased ALB levels were independently and substantially associated with renal function decline (0.11 mL/min/1.73m^2^ per year for each standard deviation fall in ALB) in the elderly, regardless of urine albumin, clinical risk factors, and assessed inflammatory markers [14]. Another Australian study showed that serum albumin was associated with an annual decline in eGFR and renal outcomes after adjustment for relevant confounders [15]. Unfortunately, in Asian populations, no studies have simultaneously reported the association of serum albumin with renal prognosis and renal function decline. At the same time, the non-linear relationship between serum albumin and renal prognosis and renal function decline has not been reported. Therefore, this study aims to investigate the relationship between serum albumin and renal prognosis and renal function decrease. In light of this, a cohort study was designed in the Japanese CKD population.

## Methods

### Study design

The design of this investigation was a prospective cohort study. The research on chronic kidney disease outcomes in treatment and epidemiology (CKD-ROUTE), a prospective cohort study of a representative Japanese population with stage G2-G5 CKD, was the source of the data used in this investigation. The CKD stage was determined using the kidney disease: improving global outcomes (KDIGO) categorization system [16]. The study’s design details have previously been reported [17–19]. More than 1,000 people were enrolled in Tokyo Medical and Dental University Hospital and its 15 affiliated hospitals [17]. We set serum albumin at baseline as the target-independent variable, and set renal composite endpoint (dichotomous variable: progressed to the renal composite endpoint, did not progress to renal composite endpoint) and renal function decline (annual decline in eGFR, continuous variable) as the dependent variable.

### Data source

We obtained the raw data for free from the DATADRYAD database provided by Iimori S et al. From: Prognosis of chronic kidney disease with normal-range proteinuria: The CKD-ROUTE study. *PLOS ONE* 2018, 13(1):e190493 [17]. Dryad Digital Repository. Dryad (https://datadryad.org/stash) data package (doi:10.5061/dryad.kq23s). Under Dryad’s terms of service, researchers could use this data for secondary analyses without violating the authors’ rights.

### Study population

**Fig. 1:**
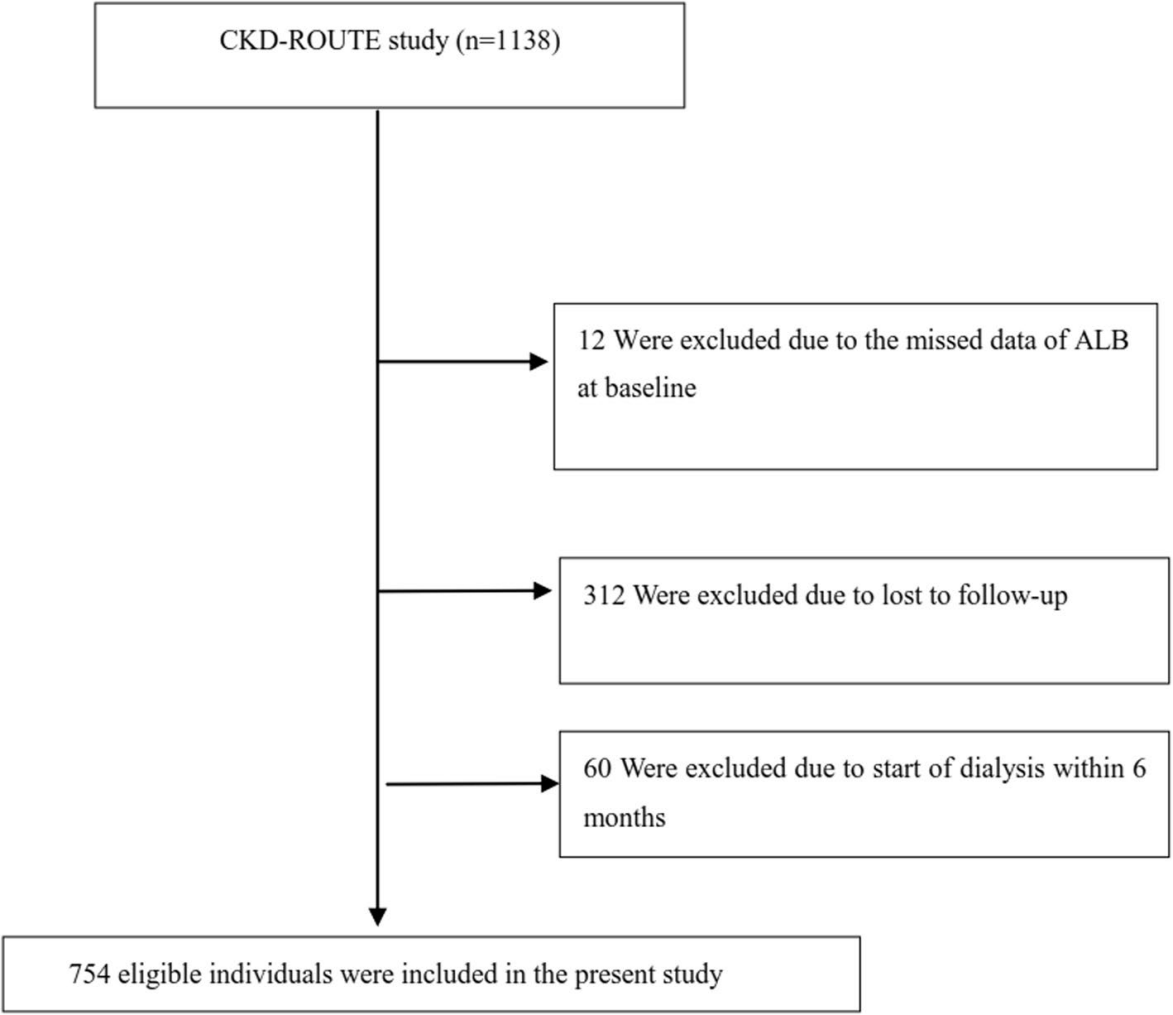
Flowchart of study participants

The original research gathered participants from the Japanese population with CKD stages G2-G5 consecutively and non-selectively. The original study included patients with stage 5 CKD because a prior study found that 35% of these patients did not receive renal replacement treatment over the 3-year observation period [20]. Between October 2010 and December 2011, new patients over the age of 20 who presented or were referred for therapy but not dialysis were recruited. All patients’ data were tracked until they reached the renal composite endpoint or 2 years after enrollment, whichever came first. Patients with malignancy, transplant recipients, or active gastrointestinal bleeding were excluded, as were those without informed consent. In the end, 1,138 patients were evaluated for eligibility in the initial study [17]. We further excluded patients with missing values of ALB (*n* = 12), lost to follow-up (*n* = 312), and patients who reached the renal composite endpoint within 6 months after enrollment (*n* = 60). This study’s final analysis included 754 participants (See Fig. 1 for details on the flowchart).

The identifying information of participants in this study was encoded into untraceable codes to minimize potential privacy concerns. This study was approved by the Ethics Committee of the Tokyo Medical and Dental University Hospital and its 15 affiliated hospitals (no. 883). After explaining the study, informed consent was obtained from all participants in this investigation [17].

### Variables

#### Serum albumin

The baseline serum albumin data was recorded as a continuous variable. Blood samples were taken for serum albumin measurement [19].

#### Definitions of outcomes

Based on previous eGFR Study results, outcomes (CKD progression) were defined [17, 21, 22] as follows: 1) Renal function decline: an annual eGFR decline (mL/min/1.73m2/year), calculated according to the slope of a least-squares plot of eGFR. 2) Renal prognosis: eGFR decreased by 50% from baseline, or dialysis initiation was considered the renal composite endpoint.

The eGFR was calculated using the renal disease equation modified for Japanese subjects and the following diet modification [23]: eGFR = 194 × serum creatinine ^−1.094^ × age ^−0.287^ (if female, × 0.739).

Participants have been monitored every 6 months. Overall, observation lasted approximately 3 years after enrollment or until the renal composite endpoint was censored, death occurred, or informed consent was withdrawn.

#### Covariates

This study’s covariates were chosen based on our clinical expertise, original research, and previous investigations of CKD progression risk factors. Therefore, based on the concepts mentioned above, the following variables were used as covariates: (1) continuous variables: body mass index (BMI), age, systolic blood pressure (SBP), eGFR, urinary protein-to-creatinine ratio (UPCR), and hemoglobin (Hb); (2) categorical variables: diabetes, gender, hypertension, history of CVD, urinary occult blood, anti-hypertensive therapy including angiotensin receptor blockers (ARB), diuretics, calcium channel blocker (CCB), and angiotensin-converting enzyme inhibitors (ACEI).

At enrolment, medical history, lifestyle behaviors (self-feeding ability), and current prescriptions were documented. Using anthropometric body height and weight measurements, the BMI was derived. The blood pressure was taken with a conventional sphygmomanometer. Urine and blood samples were collected to measure creatinine, hemoglobin, urinary protein, urinary occult blood, and urinary creatinine. Due to the Japanese health insurance system, urine albumin was not frequently measured. Therefore, UPCR was measured. Nephrotic syndrome proteinuria was defined as UPCR ≥ 3.5 g/gCr [24]. Low BMI (< 23.5 kg/m2) was defined as a cut-off value [21].

#### Definition of hypertension, cardiovascular disease, diabetes, and etiology of kidney disease

SBP of at least 140 mmHg or DBP of at least 90 mmHg, or clinician-diagnosed hypertension, or currently taking anti-hypertensive medication, was considered hypertension [17]. HbA1c 6.5% (National Glycohemoglobin Standardization Program (NGSP) standard) or antidiabetic therapy history was used to define diabetes mellitus [17, 19]. The physician treating each patient at enrollment identified each patient’s etiology of CKD based on the patient’s clinical characteristics, medical history, and histological results on renal biopsy specimens [17, 19].

History of coronary heart disease (including angina pectoris, myocardial infarction, and coronary revascularization), congestive heart failure, peripheral arterial disease, or stroke (transient ischemic attack, cerebral infarction, subarachnoid hemorrhage, or cerebral hemorrhage) was considered as CVD [17].

### Handling of missing data

There were 13(1.72%), 74(9.81%), 7(0.93%), and 45(5.97%) participants with missing data for SBP, BMI, urinary occult blood, and UPCR, respectively. To minimize bias caused by missing data on covariants, multiple imputations were performed to handle missing data on covariants [25]. The imputation model included BMI, SBP, age, etiology of CKD, gender, diabetes, use of RAAS inhibitor, UPCR, hypertension, Scr, eGFR, urinary occult blood, Hb, history of CVD, use of CCB, and use of diuretics. Missing-at-random (MAR) assumptions were used in missing data analysis procedures [26].

### Statistical analysis

We classified the individuals by ALB level quartiles. Continuous variables were represented by mean (standard deviation) (Gaussian distribution) or median (interquartile ranges) (Skewed distribution), while frequencies and percentages represented categorical variables. Using the One-Way ANOVA test (normal distribution), χ2 (categorical variables), or Kruskal–Wallis H test (skewed distribution), the differences between the various ALB groups were determined. The Kaplan–Meier method was used to calculate survival estimates and time-to-renal composite endpoints. We compared the Kaplan–Meier likelihood of renal composite endpoint-free survival among ALB groups using a log-rank test [27].

We created three models using univariate and multivariate Cox proportional-hazards and linear regression to examine the relationship between serum albumin and renal prognosis or renal function decline. Effect sizes (HR and β) were recorded, along with 95% confidence intervals [28]. Covariances should be adjusted when they change the hazard ratio by 10% or more [29]. As described by Fine and Gray, competing risks multivariate Cox’s regression was done using death as the competing hazard for the renal composite outcome [30, 31]. In this study, a subdistribution HR (SHR) was calculated with a 95% confidence interval (CI).

The non-linearity between serum albumin and the renal prognosis was addressed using a Cox proportional hazards regression model with cubic spline functions and smooth curve fitting (penalized spline method) [32]. Simultaneously, a generalized additive model (GAM) and smooth curve fitting (penalized spline method) were used to investigate the exact shape of the relationship between ALB and renal function decline [33]. After detecting non-linearity, we calculated the inflection point using a recursive algorithm. A log-likelihood ratio was used to determine the best model to describe the relationship between ALB and renal function decline and prognosis [34].

A stratified Cox proportional-hazards regression model and a linear regression model were used to perform subgroup analyses across various subgroups (gender, BMI, urinary occult blood, use of calcium channel blocker, age, CKD stage, use of diuretics, UPCR, SBP, etiology of CKD, diabetes, history of CVD, hypertension, and use of RAAS inhibitor). First, we converted the continuous variable age (< 60, ≥ 60 years) [35], BMI (< 23.5, ≥ 23.5 kg/m^2^), SBP (< 140, ≥ 140 mmHg), UPCR(< 3.5, ≥ 3.5 g/gCr) to a categorical variable based on the clinical cut point. Second, the likelihood ratio test was used to test for interaction in models with and without interaction terms [36, 37].

We conducted a series of sensitivity analyses to assess the robustness of our findings. We converted serum albumin into a categorical variable based on quartiles. To validate the results for serum albumin as a continuous variable and to investigate the possibility of non-linearity, we calculated P for the trend. Patients with diabetic nephropathy [38] and decreased renal function at baseline had an obviously increased hazard of CKD progression [39]. As a result, when investigating the relationship between ALB and CKD progression in other sensitivity analyses, we excluded participants with diabetic nephropathy or baseline eGFR < 15 ml/min per 1.73 m^2^. Besides, to ensure the robustness of the results, we used a GAM to insert the continuity covariate into the equation as a curve [40]. We also calculated E-values to investigate the possibility of unmeasured confounding between ALB and renal prognosis and renal function decline [41]. The STROBE statement was used to write the results [29].

The statistical software packages R (http://www.R-project.org, The R Foundation) and EmpowerStats (http://www.empowerstats.com, X&Y Solutions, Inc, Boston, MA) were used for modeling. Statistical significance was defined as *P* values less than 0.05 (two-sided).

## Results

### Participants’ baseline characteristics

Table 1 summarized the baseline demographic and clinical characteristics of study participants. At baseline, the population had a mean age of (66.86 ± 13.41) years old, with 522 (69.23%) being male. The mean baseline ALB and eGFR was 3.89 ± 0.59 g/dL and 33.43 ± 17.97 ml/min/1.73 m^2^. The annual eGFR decline was 2.65 mL/min/1.73 m2/year. 218 people (28.9%) experienced renal composite endpoint during a median follow-up time of 36.0 months. The most extended follow-up period was 39 months. We divided the participants into subgroups based on their ALB quartiles (< 3.6, 3.6–4.0, 4.0–4.3, ≥ 4.3 g/dL). There was no statistical difference in baseline characteristics of ALB (quartile) groups in terms of BMI, age, and use of RAAS inhibitor (*P*-value > 0.05). When we set the Q1(ALB < 3.6 g/dL) group as a reference, the higher value or proportion of Hb, eGFR, CKD stage 2–3, and etiology of CKD as nephrosclerosis were detected in the Q4 (Hb ≥ 4.3 g/dL) group, while the lower value and proportion of age, UCPR, SBP, urinary occult blood, use of diuretics, Scr, hypertension, diabetes, use of CCB, history of CVD, CKD stage 4–5, and etiology of CKD as diabetic nephropathy were observed.

**Table 1:** Baseline characteristics of all the patients at enrollment (*n* = 754)

**ALB (g/dL)**	**Q1 (< 3.6)**	**Q2 (3.6–4.0)**	**Q3 (4.0–4.3)**	**Q4 (≥ 4.3)**	***P*** **-value**
**Participants**	182	173	173	226	
**Age (years)**	66.93 ± 13.24	69.56 ± 12.67	67.91 ± 12.54	63.93 ± 14.23	< 0.001
**SBP (mmHg)**	145.47 ± 24.65	138.88 ± 20.75	137.63 ± 20.62	137.68 ± 21.23	< 0.05
**BMI (kg/m**^**2**^**)**	23.77 ± 4.07	23.72 ± 3.84	23.87 ± 3.50	23.70 ± 4.00	0.97
**HB (g/dL)**	10.86 ± 2.03	11.82 ± 1.94	12.48 ± 2.08	13.18 ± 1.97	< 0.001
**ALB (g/dL)**	3.05 ± 0.43	3.77 ± 0.11	4.10 ± 0.09	4.48 ± 0.19	< 0.001
**Scr (mg/dL)**	2.04 (1.59–3.20)	1.85 (1.29–2.70)	1.64 (1.22–2.55)	1.30 (1.07–1.89)	< 0.001
**eGFR (ml/min per 1.73 m2)**	27.57 ± 16.65	30.32 ± 16.94	33.09 ± 16.63	40.77 ± 18.37	< 0.001
**UPCR (g/gCr)**	4.15 (1.69–6.95)	0.97 (0.18–2.52)	0.54 (0.11–1.44)	0.20 (0.05–0.72)	< 0.001
**Gender**					0.71
**Male**	126 (69.23%)	115 (66.47%)	125 (72.25%)	156 (69.03%)	
**Female**	56 (30.77%)	58 (33.53%)	48 (27.75%)	70 (30.97%)	
**Etiology of CKD**					< 0.001
**Diabetic nephropathy, n(%)**	87 (47.80%)	43 (24.86%)	32 (18.50%)	20 (8.85%)	
**Nephrosclerosis, n (%)**	42 (23.08%)	65 (37.57%)	85 (49.13%)	113 (50.00%)	
**Glomerulonephritis, n (%)**	33 (18.13%)	36 (20.81%)	34 (19.65%)	44 (19.47%)	
**Other, n (%)**	20 (10.99%)	29 (16.76%)	22 (12.72%)	49 (21.68%)	
**Urinary occult blood, n(%)**	95 (52.20%)	48 (27.75%)	51 (29.48%)	49 (21.68%)	< 0.001
**Hypertension, n (%)**	176 (96.70%)	159 (91.91%)	156 (90.17%)	192 (84.96%)	< 0.001
**History of CVD, n (%)**	62 (34.07%)	43 (24.86%)	47 (27.17%)	41 (18.14%)	< 0.05
**Diabetes, n (%)**	99 (54.40%)	66 (38.15%)	57 (32.95%)	55 (24.34%)	< 0.001
**Use of RAAS inhibitor, n(%)**	131 (71.98%)	117 (67.63%)	119 (68.79%)	137 (60.62%)	0.09
**Use of calcium channel blocker, n (%)**	109 (59.89%)	93 (53.76%)	81 (46.82%)	98 (43.36%)	< 0.05
**Use of diuretics, n (%)**	90 (49.45%)	56 (32.37%)	41 (23.70%)	52 (23.01%)	< 0.001
**CKD stage, n (%)**					< 0.001
**2**	12 (6.59%)	11 (6.36%)	8 (4.62%)	31 (13.72%)	
**3**	53 (29.12%)	66 (38.15%)	86 (49.71%)	122 (53.98%)	
**4**	70 (38.46%)	66 (38.15%)	53 (30.64%)	61 (26.99%)	
**5**	47 (25.82%)	30 (17.34%)	26 (15.03%)	12 (5.31%)	

Continuous variables are presented as mean ± standard deviation and median with interquartile ranges. Categorical data are presented as numbers and percentages

*Abbreviations*: *SBP* Systolic blood pressure, *BMI* Body mass index, *Scr* Serum creatinine, *HB* Hemoglobin, *ALB* Serum albumin, *CKD* Chronic kidney disease, *eGFR* Estimated glomerular filtration rate, *CVD* Cardiovascular disease, *UPCR* Urinary protein/creatinine ratio, *g/gCr* Gram per gram creatinine, *RAAS* Renin–angiotensin–aldosterone system

Patients were divided into two groups based on whether or not they had a renal composite endpoint. The ALB levels in the two groups were shown in Fig. S1. The results revealed that the level of ALB was lower in the composite renal endpoint group.

In our study, 372 patients were excluded because of a loss of follow-up. We compared patients who stayed in the final analysis with patients who were lost to follow-up. Table S1 showed no statistical difference in baseline characteristics in terms of BMI, SBP, eGFR, UPCR, gender, etiology of CKD, urinary occult blood, hypertension, diabetes, and history of CVD (*P*-value > 0.05). When we set the not lost to follow-up group as a reference, the higher value and proportion of age and renal composite endpoint were detected in the lost to follow-up group, while the lower value of ALB and Hb were observed.

### The annual eGFR decline and incidence rate of the renal composite endpoint

**Fig. 2:**
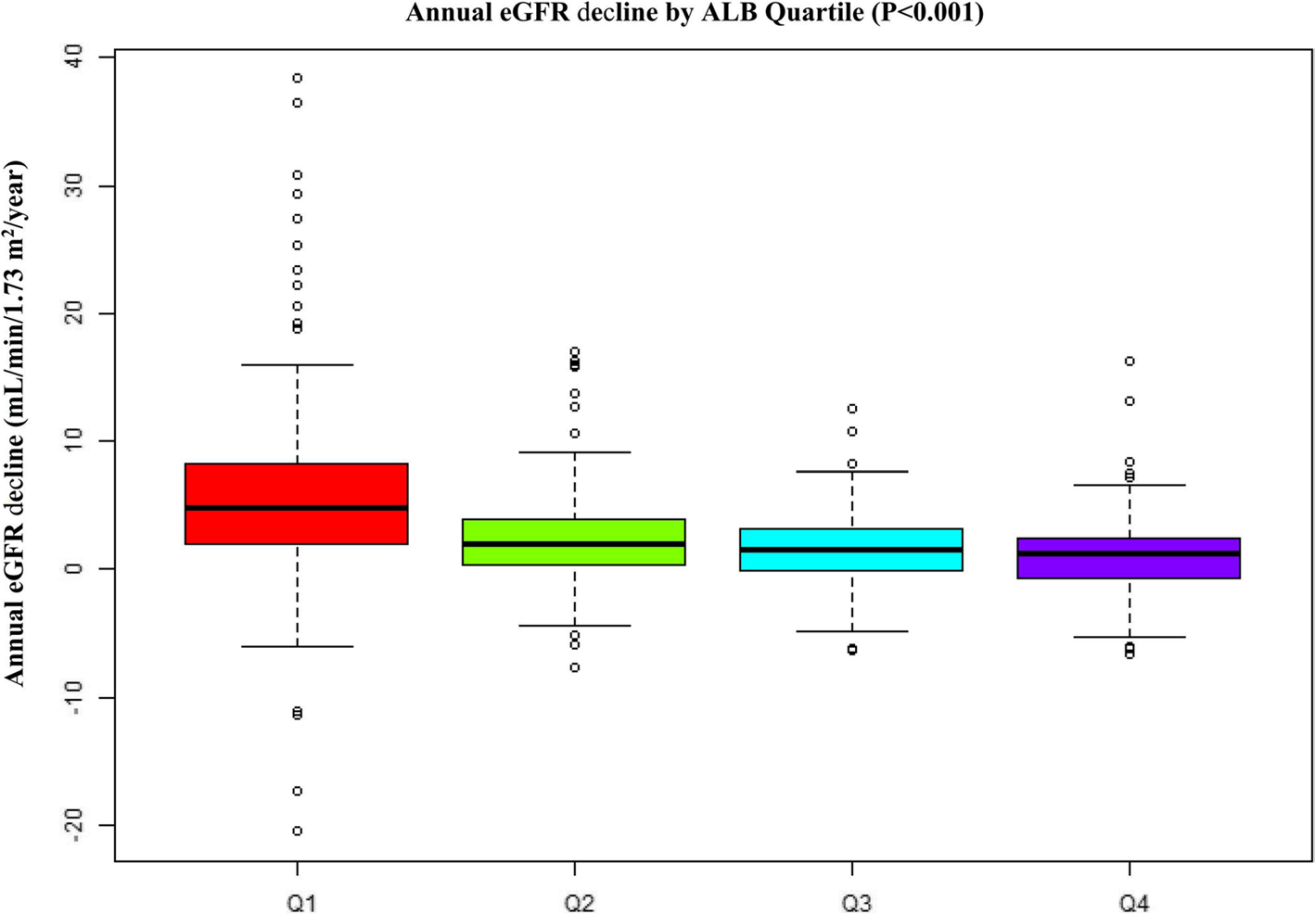
Average annual eGFR decline according to the quartile of serum albumin

**Fig. 3:**
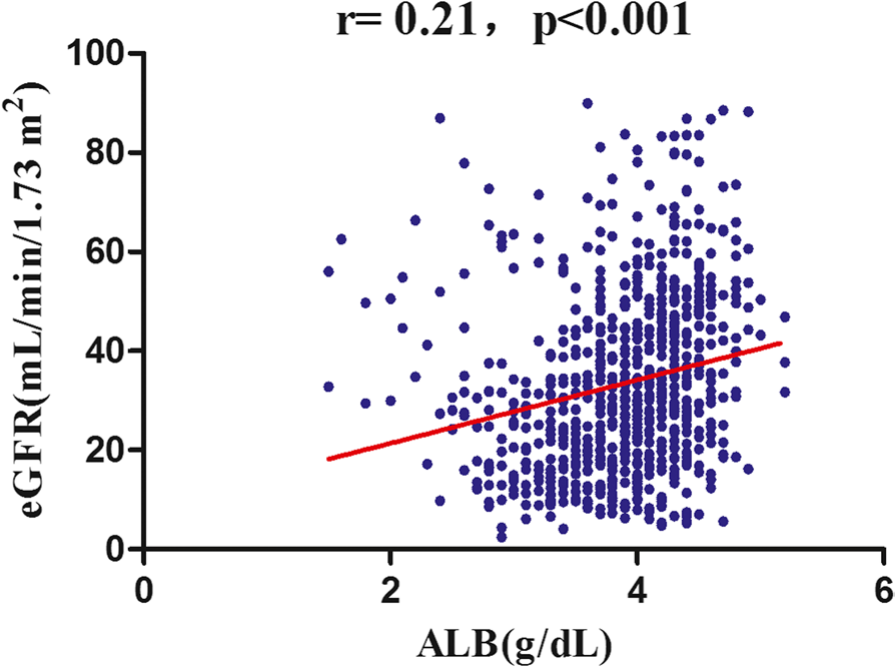
Correlation analysis between serum albumin and eGFR

**Fig. 4:**
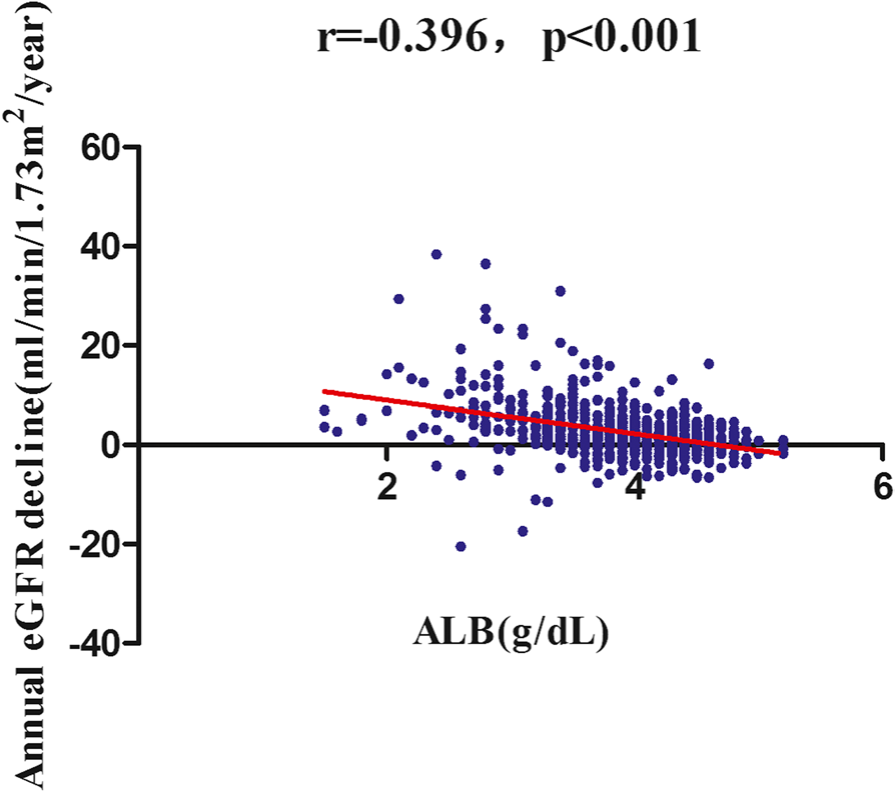
Correlation analysis between serum albumin and the annual decline in eGFR

During a median follow-up period of 36.0 months, 218 (28.9%) patients developed renal composite endpoints, as shown in Table 2. We displayed the number compositions of renal outcomes across different groups of quartiles of ALB in different time points (0–12 months, 12–24 months, and >  = 24 months). The results showed that the patients with a higher ALB had a lower incidence of the renal composite endpoint no matter in which time points (*P* < 0.001 for trend). We also found that those with a higher ALB had a lower annual eGFR decline (*P* < 0.001) (Fig. 2). Correlation analysis also found that baseline serum albumin levels were positively correlated with baseline eGFR (*r* = 0.21 *p* < 0.001) (Fig. 3), and inversely correlated with the annual decline in eGFR (*r* = -0.40, *p* < 0.001) (Fig. 4).

**Table 2:** Incidence rate of the renal composite endpoint

**ALB**	**Participants (n)**	**Renal composite endpoint, n (%)**			**Death, n(%)**
		**0–12 months**	**12-24 months**	** > = 24 months**	
**Total**	754	47 (6.23)	77 (10.21)	94 (12.47)	15 (1.99)
**Q1**	182	32 (17.58)	45 (24.73)	37 (20.33)	5 (2.75)
**Q2**	173	9 (5.20)	16 (9.25)	26 (15.03)	2 (1.16)
**Q3**	173	3 (1.73)	10 (5.78)	19 (10.98)	2 (1.16)
**Q4**	226	3 (1.33)	6 (2.65)	12 (5.31)	6 (2.65)
***P*** **for trend**		< 0.001	< 0.001	< 0.001

Male respondents had a higher incidence of the renal composite endpoint than female subjects when the age was less than 60 in the age stratification by 20 intervals (Fig. S2). In contrast, male subjects exhibited a lower incidence of the renal composite endpoint than female subjects when their ages were between 60 and 80. It should be pointed out that regardless of age group, male patients have a higher annual decline in eGFR than female patients (Fig. S3).

We also analyzed the mortality rates for each group of patients. The incidence of total mortality and each ALB group was 1.99%, 2.75%, 1.16%, 1.16%, and 2.65%, respectively.

### The results of univariate analysis using the linear regression model and the Cox proportional-hazards regression model

The available data were subjected to a univariate analysis, which revealed that the factors of age, gender, BMI, and CVD history were not related to the renal composite endpoint (all *P*-values > 0.05). However, SBP, urinary occult blood, Scr, UPCR, diabetes, hypertension, use of calcium channel blocker, use of RAAS inhibitor, use of diuretics, and CKD stage 4–5 were positively linked to CKD progression. And Hb, eGFR, and ALB were negatively connected with CKD prognosis (See Table 3 for detail).

**Table 3:** The results of univariate analysis

**Variable**	**Incident renal composite endpoint**		**Annual eGFR decline (mL/min/1.73 m2/year)**	
	**HR (95%CI)**	***P*** ** value**	**β (95% CI)**	***P*** ** value**
**Age (years)**	0.99 (0.98, 1.00)	0.051	-0.03 (-0.05, -0.00)	< 0.05
**Gender**				
**Male**	Ref		Ref	
**Female**	0.99 (0.74, 1.32)	0.94	-1.00 (-1.78, -0.23)	< 0.05
**Etiology of CKD**				
**Diabetic nephropathy,**	Ref		Ref	
**Nephrosclerosis,**	0.20 (0.14, 0.28)	< 0.001	-3.86 (-4.72, -2.99)	< 0.001
**Glomerulonephritis**	0.32 (0.22, 0.46)	< 0.001	-3.01 (-4.04, -1.99)	< 0.001
**Other**	0.21 (0.13, 0.33)	< 0.001	-4.64 (-5.73, -3.55)	< 0.001
**BMI (kg/m**^**2**^**)**	1.02 (0.99, 1.06)	0.19	-0.01 (-0.10, 0.08)	0.85
**SBP (mmHg)**	1.02 (1.01, 1.02)	< 0.001	0.05 (0.04, 0.07)	< 0.001
**HB (g/dL)**	0.70 (0.66, 0.75)	< 0.001	-0.28 (-0.45, -0.12)	< 0.001
**Scr (mg/dL)**	1.43 (1.37, 1.49)	< 0.001	0.21 (-0.04, 0.46)	0.11
**eGFR (ml/min per 1.73 m2)**	0.92 (0.91, 0.93)	< 0.001	-0.02 (-0.04, 0.002)	0.07
**ALB (g/dL)**	0.34 (0.29, 0.41)	< 0.001	-3.37 (-3.93, -2.81)	< 0.001
**Urinary occult blood**				
**No**	Ref		Ref	
**Yes**	1.57 (1.20, 2.06)	< 0.05	1.68 (0.92, 2.43)	< 0.001
**UPCR(g/gCr)**	1.20 (1.17, 1.23)	< 0.001	0.74 (0.64, 0.84)	< 0.001
**Hypertension**				
**No**	Ref		Ref	
**Yes**	4.06 (1.80, 9.13)	< 0.001	1.81 (0.59, 3.03)	< 0.05
**History of CVD**				
**No**	Ref		Ref	
**Yes**	1.25 (0.93, 1.68)	0.13	0.33 (-0.49, 1.15)	0.43
**Diabetes**				
**No**	Ref		Ref	
**Yes**	2.49 (1.90, 3.25)	< 0.001	1.78 (1.05, 2.51)	< 0.001
**Use of RAAS inhibitor**				
**No**	Ref		Ref	
**Yes**	1.72 (1.26, 2.36)	< 0.001	1.50 (0.75, 2.26)	< 0.001
**Use of calcium channel blocker**				
**No**	Ref		Ref	
**Yes**	1.73 (1.32, 2.28)	< 0.001	0.61 (-0.11, 1.32)	0.10
**Use of diuretics**				
**No**	Ref		Ref	
**Yes**	2.19 (1.68, 2.86)	< 0.001	1.55 (0.79, 2.32)	< 0.001
**CKD stage**				
**2**	Ref		Ref	
**3**	1.63 (0.58, 4.58)	0.35	-0.41 (-1.77, 0.95)	0.55
**4**	6.27 (2.30, 17.06)	< 0.001	0.37 (-1.02, 1.77)	0.60
**5**	21.88 (8.02, 59.68)	< 0.001	0.41 (-1.14, 1.96)	0.61

Univariate linear regression model results showed that BMI, eGFR, Scr, history of CVD, use of CCB, and CKD stage were not related to annual eGFR decline (All *P*-value > 0.05). Still, age, female, Hb, and ALB were negatively connected with renal function decline. And SBP, urinary occult blood, UPCR, hypertension, diabetes, use of RAAS inhibitors, use of diuretics were positively linked to renal function decline. Patients with primary onset diabetic nephropathy were also found to have a high hazard of renal composite endpoint and renal function decline (Table 3).

**Fig. 5:**
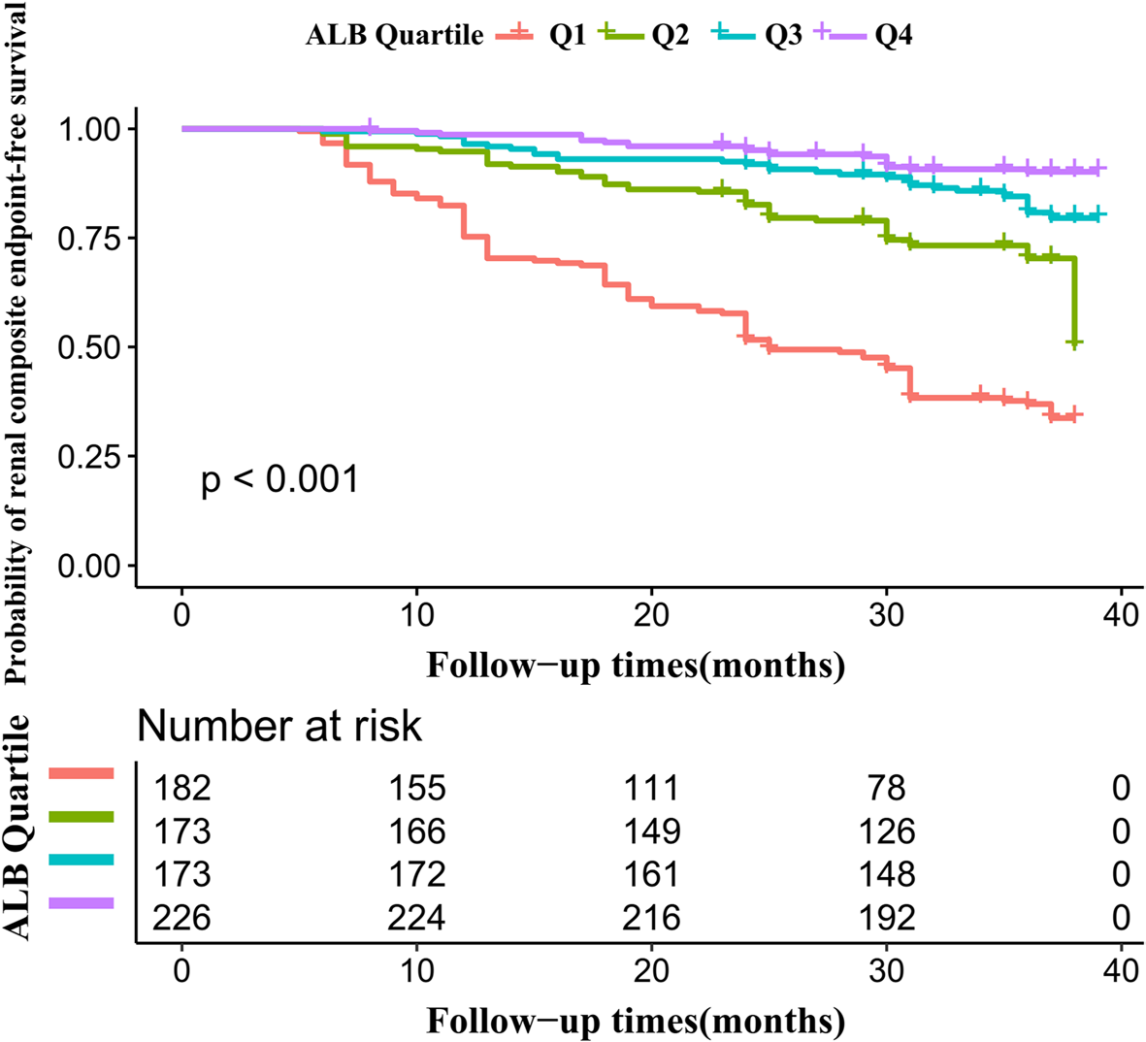
Kaplan–Meier event-free survival curve

The Kaplan–Meier survival curves for renal composite endpoint-free survival probability stratified by ALB groups were depicted in Fig. 5. The likelihood of renal composite endpoint-free survival differed significantly between ALB groups (log-rank test, *p* < 0.001). With increasing ALB, renal composite endpoint-free survival probabilities increased, indicating that the lowest endpoint hazard was among the top groups.

### Multivariate analysis results using the Cox proportional-hazards regression model

As ALB met the proportional hazards assumption, the association between the ALB and the renal composite endpoint was evaluated by the Cox proportional hazards regression model. In the unadjusted model (crude model), a 1 g/dL increase in serum albumin was associated with a 66% reduction in the hazard of the renal composite endpoint (HR = 0.34, 95%CI 0.29 to 0.41). When we only adjusted for demographic variables in the minimally-adjusted model (model I), each additional g/dL of serum albumin increase reduced the hazard of the renal composite endpoint by 56% (HR = 0.44, 95%CI 0.36 to 0.54). Each additional g/dL of serum albumin was associated with a 39% decrease in renal composite endpoint (HR = 0.61, 95%CI 0.45 to 0.82) in the fully adjusted model (model II) (Table 4).

**Table 4:** Relationship between ALB and the renal composite endpoint in different models

**Exposure**	**Crude model (HR,95%CI, ** ***P*** **)**		**Model I (HR,95%CI, ** ***P*** **)**		**Model II (HR,95%CI, ** ***P*** **)**		**Model III (HR,95%CI, ** ***P*** **)**	
**ALB**	0.34 (0.29, 0.41)	< 0.001	0.44 (0.36, 0.54)	< 0.001	0.61 (0.45, 0.81)	< 0.001	0.68 (0.50, 0.92)	< 0.05
**ALB Quartile**								
**Q1**	Ref		Ref		Ref		Ref	
**Q2**	0.34 (0.25, 0.48)	< 0.001	0.44 (0.31, 0.62)	< 0.001	0.70 (0.47, 1.02)	0.06	0.71 (0.48, 1.05)	0.08
**Q3**	0.19 (0.13, 0.29)	< 0.001	0.27 (0.18, 0.41)	< 0.001	0.45 (0.29, 0.70)	< 0.001	0.50 (0.31, 0.79)	< 0.05
**Q4**	0.10 (0.06, 0.15)	< 0.001	0.15 (0.09, 0.25)	< 0.001	0.43 (0.25, 0.73)	< 0.05	0.65 (0.38, 1.13)	0.13
***P*** ** for trend**	< 0.001		< 0.001		< 0.001		< 0.05	

Crude model: we did not adjust other covariants

Model I: we adjust gender, age, BMI, hypertension, SBP, history of CVD, diabetes, and etiology of CKD

Model II: we adjust age, BMI, gender, SBP, diabetes, hypertension, etiology of CKD, use of calcium channel blocker, UPCR, history of CVD, eGFR, urinary occult blood, Hb, use of RAAS inhibitor, use of diuretics

Model III: we adjust age(smooth), gender, SBP(smooth), BMI(smooth), hypertension, diabetes, history of CVD, Hb(smooth), UPCR(smooth), etiology of CKD, eGFR(smooth), use of RAAS inhibitor, urinary occult blood, use of calcium channel blocker, use of diuretics

*CI* Confidence, *Ref* Reference, *HR* Hazard ratios

### The results of competing risks multivariate Cox’s regression

There were 15 (1.99%) cases that died before the renal outcome. Table 5 displayed the competing analysis results when death was treated as a competing event. Serum albumin negatively affected renal prognosis in the crude model (SHR = 0.34, 95% CI:0.29 to 0.41). In the minimally adjusted model, the result did not have a noticeable change (SHR:0.44, 95%CI: 0.36–0.54). We could also detect the link (SHR = 0.60, 95%CI: 0.45 to 0.81) in the fully adjusted model (model II). This result was similar to when the competing hazard of death was not considered.

**Table 5:** Relationship between ALB and chronic kidney disease progression in different models with the competing risk of mortality

**Exposure**	**Crude model (SHR,95%CI, ** ***P*** **)**		**Model I (SHR,95%CI, ** ***P*** **)**		**Model II (SHR,95%CI, ** ***P*** **)**	
**ALB**	0.34 (0.29, 0.41)	< 0.001	0.44 (0.36, 0.54)	< 0.001	0.60 (0.45, 0.81)	< 0.001
**ALB Quartile**						
**Q1**	Ref		Ref		Ref	
**Q2**	0.34 (0.25, 0.48)	< 0.001	0.44 (0.31, 0.62)	< 0.001	0.69 (0.47, 1.01)	0.06
**Q3**	0.19 (0.13, 0.29)	< 0.001	0.27 (0.18, 0.41)	< 0.001	0.45 (0.29, 0.70)	< 0.001
**Q4**	0.10 (0.06, 0.15)	< 0.001	0.15 (0.09, 0.25)	< 0.001	0.42(0.25, 0.72)	< 0.05
***P*** ** for trend**	< 0.001		< 0.001		< 0.001	

Crude model: we did not adjust other covariants

Model I: we adjust age, BMI, gender, hypertension, SBP, history of CVD, diabetes, and etiology of CKD

Model II: we adjust age, BMI, gender, SBP, diabetes, hypertension, etiology of CKD, use of calcium channel blocker, UPCR, history of CVD, eGFR, urinary occult blood, Hb, use of RAAS inhibitor, use of diuretics

*CI* Confidence, *Ref* Reference, *SHR* Subdistribution hazard ratios

### The results of multivariate analyses utilizing the linear regression model

We created three linear regression models to investigate the relationship between serum albumin and renal function decline. An increase of 1 g/dL of serum albumin was associated with 3.37 mL/min/1.73 m^2^/year decreases in annual eGFR decline (β = -3.37, 95%CI -3.93 to -2.82) in the unadjusted model (crude model). We only adjusted for demographic variables using the minimally-adjusted model (model I). Each additional g/dL of serum albumin increase could result in an annual eGFR decline of 2.65 mL/min/1.73 m^2^/year (β = -2.65, 95%CI -3.22 to -2.07). Each additional g/dL of serum albumin was associated with a 1.41 mL/min/1.73 m^2^/year decrease in annual eGFR decline (β = -1.41, 95%CI -2.11 to -0.72) in the fully adjusted model (model II) (Table 6).

**Table 6:** Relationship between ALB and renal function decline in different models

**Exposure**	**Crude model (β,95%CI, ** ***P*** **)**		**Model I (β,95%CI, ** ***P*** **)**		**Model II (β,95%CI, ** ***P*** **)**		**Model III (β,95%CI, ** ***P*** **)**	
**ALB**	-3.37 (-3.93, -2.82)	< 0.001	-2.65 (-3.22, -2.07)	< 0.001	-1.41 (-2.11, -0.72)	< 0.001	-1.13 (-1.83, -0.43)	< 0.05
**ALB Quartile**								
**Q1**	Ref		Ref		Ref		Ref	
**Q2**	-3.50 (-4.47, -2.53)	< 0.001	-2.59 (-3.54, -1.65)	< 0.001	-1.19 (-2.19, -0.19)	< 0.05	-1.10 (-2.10, -0.10)	< 0.05
**Q3**	-4.373 (-5.34, -3.40)	< 0.001	-3.37 (-4.33, -2.41)	< 0.001	-1.70 (-2.76, -0.64)	< 0.05	-1.35 (-2.41, -0.29)	< 0.05
**Q4**	-4.811 (-5.72, -3.90)	< 0.001	-3.60 (-4.55, -2.66)	< 0.001	-1.91 (-2.99, -0.83)	< 0.001	-1.49 (-2.58, -0.41)	< 0.05
***P*** ** for trend**	< 0.001		< 0.001		< 0.001		< 0.05	

Crude model: we did not adjust other covariants

Model I: we adjust age, history of CVD, BMI, gender, SBP, diabetes, hypertension, and etiology of CKD

Model II: we adjust age, BMI, gender, SBP, diabetes, use of calcium channel blocker, hypertension, etiology of CKD, UPCR, history of CVD, eGFR, urinary occult blood, Hb, use of RAAS inhibitor, use of diuretics

Model III: we adjust age (smooth), etiology of CKD, gender, history of CVD, use of RAAS inhibitor, eGFR (smooth), SBP (smooth), BMI (smooth), hypertension, diabetes, UPCR (smooth), use of calcium channel blocker, Hb (smooth), urinary occult blood, use of diuretics

*CI* Confidence, *Ref* Reference

### Sensitivity analysis

A series of sensitivity analyses were carried out to confirm the robustness of our findings. Serum albumin was first transformed from a continuous variable to a categorical variable (according to quartile) and then added back into the model. The results demonstrated that when serum albumin was converted into a categorical variable, the trend of the effect sizes (HR, SHR, or β) in various groups was equal, and P for the trend was consistent with the finding when serum albumin was a continuous variable (Tables 4, 5 and 6).

In addition, we used a GAM to insert the continuity covariate into the equation as a curve. This was essentially consistent with the fully adjusted model, according to the Model III results in Tables 4 and 6 (HR = 0.68, 95%CI: 0.50–0.92, *P* < 0.05) and (β = -1.13, 95%CI: -1.83 to-0.43, *P* < 0.05). Besides, we calculated an E-value to evaluate the sensitivity to unmeasured confounding. HR and β had E-values of 2.17 and 3.26, respectively. The E-value was higher than the relative hazard of unmeasured confounders and serum albumin, implying that unmeasured or unknown confounders had little influence on the relationship between serum albumin and renal prognosis and renal function decline.

Furthermore, we excluded patients with primary onset diabetic nephropathy in other sensitivity analyses. After controlling for confounding factors, serum albumin was found to be negatively associated with the renal composite endpoint (HR = 0.48, 95% CI:0.32 to 0.74) and the annual eGFR decline (β = -1.03, 95% CI: -1.72 to -0.35) (Table 7). For sensitivity analyses, we also excluded patients with baseline eGFRs of less than 15 ml/min per 1.73 m^2^. The results suggested that serum albumin was still negatively associated with renal composite endpoint (HR = 0.55, 95%CI:0.39 to 0.78) and the annual eGFR decline (β = -1.38, 95% confidence interval (CI): -2.15 to -0.63) (Table 7). The results of all sensitivity analyses demonstrated the robustness of our findings.

**Table 7:** Relationship between ALB and renal composite endpoint and renal function decline in different sensitivity analyses

**Exposure**	**Model I (HR, 95%CI, ** ***P*** **)**		**Model II (β, 95%CI, ** ***P*** **)**	
**eGFR ≥ 15(ml/min per 1.73 m2)**				
**ALB**	0.55 (0.39, 0.78)	< 0.001	-1.38 (-2.15, -0.60)	< 0.001
**ALB Quartile**				
**Q1**	Ref		Ref	
**Q2**	0.52 (0.31, 0.86)	< 0.05	-1.29 (-2.46, -0.11)	< 0.05
**Q3**	0.31 (0.16, 0.57)	< 0.001	-1.79 (-3.03, -0.56)	< 0.05
**Q4**	0.27 (0.14, 0.53)	< 0.001	-1.95 (-3.18, -0.72)	< 0.05
***P*** ** for trend**	< 0.001		< 0.05	
**Without DN**				
**ALB**	0.48 (0.32, 0.74)	< 0.001	-1.03 (-1.72, -0.35)	< 0.05
**ALB Quartile**				
**Q1**	Ref		Ref	
**Q2**	0.65 (0.39, 1.10)	0.11	-0.48 (-1.51, 0.56)	0.37
**Q3**	0.52 (0.29, 0.93)	< 0.05	-0.75 (-1.82, 0.32)	0.17
**Q4**	0.42 (0.22, 0.78)	< 0.05	-1.03 (-2.10, 0.03)	0.06
***P*** ** for trend**	< 0.05		0.05	

Model I was a sensitivity analysis of the relationship between ALB and CKD progression. We adjusted gender, age, SBP, BMI, hypertension, Hb, history of CVD, UPCR, diabetes, eGFR, use of RAAS inhibitor, urinary occult blood, use of calcium channel blocker, use of diuretics

Model II was a sensitivity analysis of the relationship between ALB and kidney function decline. We adjusted age, BMI, gender, SBP, diabetes, hypertension, history of CVD, eGFR, UPCR, use of calcium channel blocker, Hb, use of RAAS inhibitor, urinary occult blood, use of diuretics

*HR* Hazard ratios, *CI* Confidence, *Ref* Reference

### The non-linear relationship

**Fig. 6:**
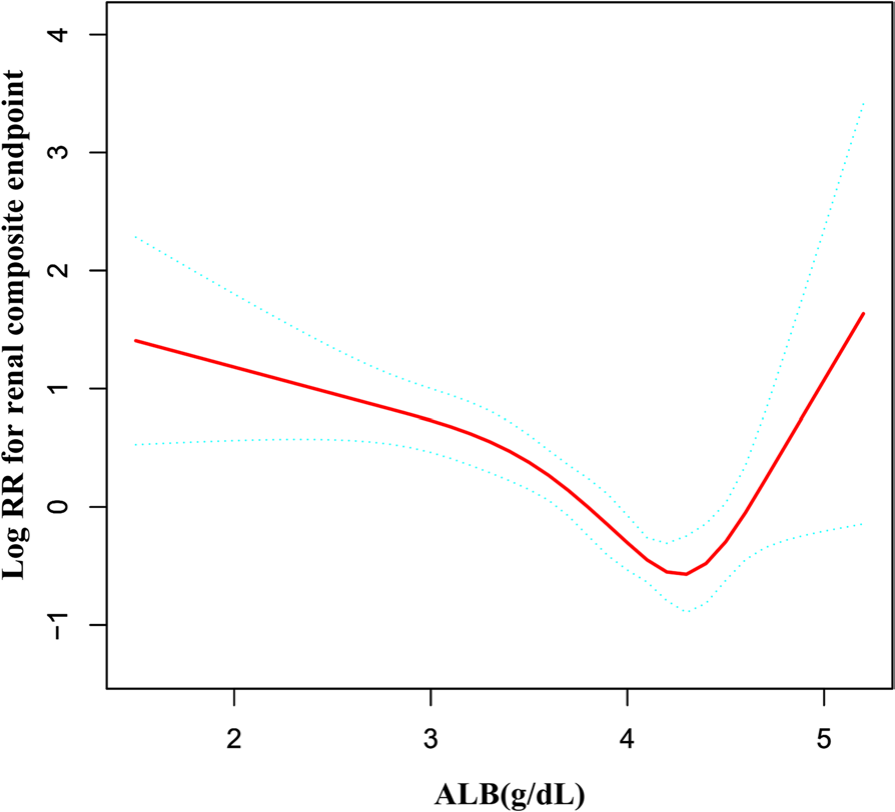
The non-linear relationship between serum albumin and the hazard of the renal composite endpoint

We discovered that the relationship between serum albumin and the renal prognosis was non-linear (Fig. 6). Using a recursive technique, we first determined an inflection point of 4.3 g/dL. When ALB was less than 4.3 g/dL, a 1 g/dL decrease in ALB levels was associated with a 58.0% increase in adjusted HR for the hazard of the renal composite endpoint (HR = 0.42, 95% CI: 0.32–0.56). However, when ALB was greater than 4.3 g/dL, a one-unit increase in ALB level was not associated with an increased hazard of the renal composite endpoint (HR = 6.11, 95% CI: 0.98–38.22) (Table 8).

**Table 8:** The result of the two-piecewise Cox regression model and linear regression model

	**Renal composite endpoint (HR, 95%CI, ** ***P*** **)**	
Fitting model by two-piecewise Cox regression		
Inflection point of ALB	4.3 g/dL	
≤ 4.3 g/dL	0.42 (0.32, 0.56)	< 0.001
> 4.3 g/dL	6.11 (0.98, 38.22)	0.05
*P* for log-likelihood ratio test	< 0.05	
	**Renal function decline (β, 95%CI, P)**	
Fitting model by two-piecewise linear regression		
Inflection point of ALB	4.1 g/dL	
≤ 4.1 g/dL	-2.79 (-3.62, -1.96)	< 0.001
> 4.1 g/dL	0.02 (-1.79, 1.84)	0.98
*P* for log-likelihood ratio test	< 0.05	

We adjusted age, BMI, gender, SBP, diabetes, hypertension, history of CVD, eGFR, UPCR, ALB, use of RAAS inhibitor, urinary occult blood, use of calcium channel blocker, and use of diuretics

*HR* Hazard ratios, *CI* Confidence interval

**Fig. 7:**
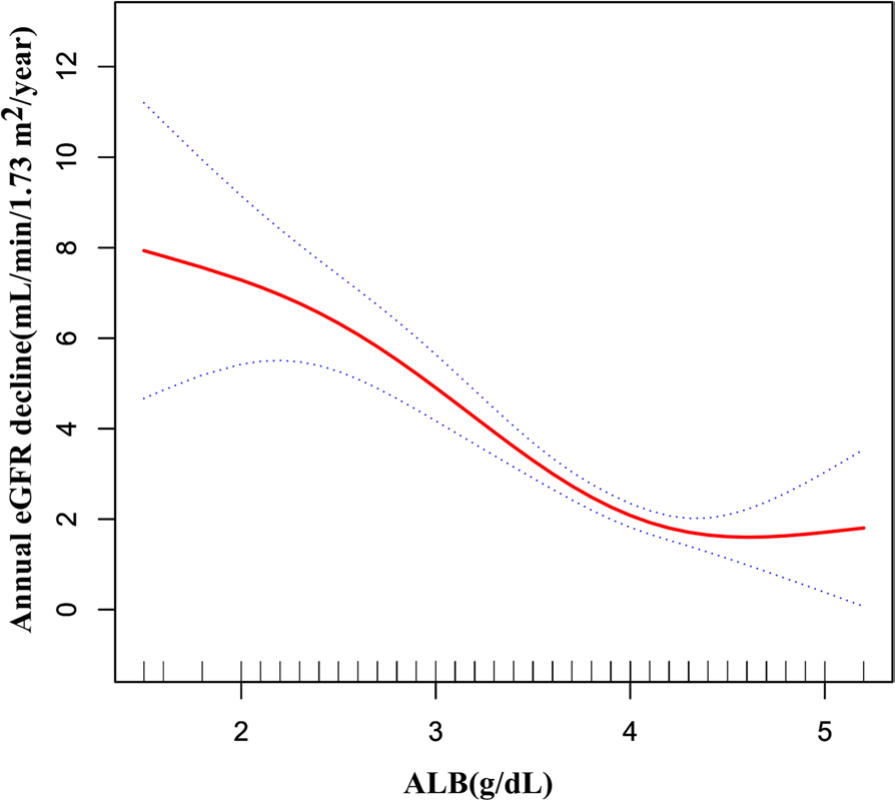
The non-linear relationship between serum albumin and the annual decline in eGFR

After controlling for confounding factors, the relationship between serum albumin and the annual decline in eGFR was found to be non-linear (Fig. 7). We calculated the inflection point to be 4.1 g/dL. We observed a strong negative association between serum albumin and the annual decline in eGFR on the left side of the inflection point, the β and 95%CI were -2.79, -3.62 to -1.96, respectively. On the right side of the inflection point, we observed a non-significant relationship between serum albumin and the annual decline in eGFR. The β and 95%CI were 0.02, -1.79 to 1.84, respectively (Table 8).

### The results of subgroup analyses

In all of the prespecified or exploratory subgroups evaluated (Table 9), there was no significant interaction in age, BMI, hypertension, gender, use of RAAS inhibitor, UPCR, history of CVD, use of diuretics, diabetes, use of CCB, and CKD stage. On the other hand, significant interactions were found in variables such as urinary occult blood and CKD etiology.

**Table 9:** Results of subgroup analysis and interaction analysis

**Characteristic**	**No of participants**	**HR (95%CI)**	***P*** ** for interacion**	**β(95%CI)**	***P*** ** for interaction**
**Age (years)**			0.07		0.30
** < 60 (years)**	177	0.81 (0.54, 1.20)		-0.60 (-2.01, 0.81)	
** ≥ 60 (years)**	577	0.53 (0.37, 0.75)		-1.41 (-2.19, -0.63)	
**Gender**			0.21		0.11
**Male**	522	0.64 (0.47, 0.89)		-0.97 (-1.80, -0.13)	
**Female**	232	0.41 (0.22, 0.76)		-2.15 (-3.37, -0.94)	
**Urinary occult blood**			0.64		< 0.05
**No**	511	0.53 (0.34, 0.80)		-0.84 (-1.72, 0.04)	
**Yes**	243	0.60 (0.40, 0.91)		-2.37 (-3.39, -1.36)	
**BMI (kg/m2)**			0.10		0.96
** < 23.5**	381	0.45 (0.30, 0.68)		-1.61 (-2.50, -0.72)	
** ≥ 23.5**	373	0.72 (0.47, 1.10)		-1.58 (-2.57, -0.59)	
**UPCR (g/gCr)**			0.97		0.17
** < 3.5**	595	0.59 (0.38, 0.91)		-1.47 (-2.29, -0.64)	
** ≥ 3.5**	159	0.58 (0.41, 0.84)		-2.43 (-3.58, -1.27)	
**Etiology of CKD**			< 0.05		0.13
**Diabetic nephropathy**	182	0.86 (0.56, 1.32)		-1.79 (-3.17, -0.40)	
**Nephrosclerosis,**	305	0.23 (0.12, 0.44)		-1.69 (-2.82, -0.57)	
**Glomerulonephritis**	147	0.52 (0.21, 1.25)		-1.49 (-3.13, 0.15)	
**Other**	120	1.80 (0.69, 4.68)		0.29 (-1.22, 1.80)	
**Use of RAAS inhibitor**			0.63		0.22
**No**	250	0.71 (0.39, 1.31)		-1.92 (-3.02, -0.82)	
**Yes**	504	0.61 (0.43, 0.85)		-1.11 (-1.94, -0.27)	
**Hypertension**			0.42		0.85
**No**	71	0.28 (0.04, 1.97)		-1.56 (-3.82, 0.70)	
**Yes**	683	0.61 (0.45, 0.82)		-1.33 (-2.07, -0.59)	
**History of CVD**		0.42			0.94
**No**	561	0.68 (0.46, 1.00)		-1.42 (-2.24, -0.60)	
**Yes**	193	0.54 (0.36, 0.81)		-1.47 (-2.50, -0.45)	
**Diabetes**		0.09			0.13
**No**	477	0.49 (0.33, 0.72)		-1.72 (-2.56, -0.89)	
**Yes**	277	0.73 (0.51, 1.06)		-0.68 (-1.82, 0.48)	
**Use of CCB**			0.64		0.26
**No**	373	0.65 (0.45, 0.93)		-1.81 (-2.74, -0.89)	
**Yes**	381	0.58 (0.41, 0.84)		-1.09 (-2.05, -0.14)	
**Use of diuretics**			0.96		0.24
**No**	515	0.59 (0.40, 0.87)		-1.11 (-1.97, -0.26)	
**Ye**	239	0.60 (0.41, 0.89)		-1.91 (-3.00, -0.83)	
**CKD stage**			0.63		0.51
**2**	62	0.44 (0.12, 1.62)		-1.04 (-3.65, 1.56)	
**3**	327	0.56 (0.36, 0.87)		-0.61 (-1.77, 0.56)	
**4**	250	0.69 (0.48, 1.01)		-1.82 (-3.00, -0.65)	
**5**	115	0.80 (0.47, 1.35)		-1.30 (-3.20, 0.59)	

Note 1: Above model adjusted for gender, age, BMI, hypertension, history of CVD, diabetes, SBP, eGFR, UPCR, Hb, use of RAAS inhibitor, urinary occult blood, use of calcium channel blocker and use of diuretics

Note 2: In each case, the model is not adjusted for the stratification variable

Specifically, a stronger association between ALB and the renal prognosis was observed in patients with nephrosclerosis (HR = 0.23, 95%CI:0.12–0.44) and glomerulonephritis (HR = 0.52, 95%CI:0.21–1.25) as etiologies of CKD, but was attenuated in patients with diabetic nephropathy (HR = 0.86,95%CI:0.56–1.32).

Subgroup analyses also showed ALB was more strongly associated with renal function decline in patients with urinary occult blood (β = -2.37,95%CI: -3.39 to -1.36), while the weaker association was probed in participants without urinary occult blood (β = -0.84, 95%CI:-1.72 to 0.04).

## Discussion

The prospective cohort study was designed to examine the link of serum albumin on renal prognosis and renal function decline in the Japanese population with stage G2-G5 CKD. Our findings suggested that the increased serum albumin was related to a significant decrease in the hazard of renal composite endpoint and a reduction of annual eGFR decline. The result was similar to that when the competing risk of death was considered. In addition, a saturation effect curve was also found as well, and different relationships of serum albumin on renal prognosis and renal function decline were detected on both sides of the inflection point. Furthermore, urinary occult blood and CKD etiology were found as potential effect modifiers to modify the relationship between serum albumin and CKD progression.

Song et al. discovered that ALB was negatively associated with renal prognosis in 1138 patients with CKD (HR = 0.75, 95%CI: 0.56–0.98, *P* < 0.05) after adjusting for confounding variables in a recent retrospective study [42]. Another Australian study showed that serum albumin was associated with an annual decline in eGFR and renal outcomes in CKD patients after adjustment for adjusted for gender, urine albumin creatinine ratio (UACR), triglycerides, age, diabetes, C-reactive protein (CRP), total cholesterol, BMI, waist-hip ratio (WHR), and alcohol consumption. They discovered that each additional g/L of serum albumin increase could lead to the annual eGFR decline decreasing 0.31 mL/min/1.73 m^2^/year (β = -0.31, 95%CI -0.18 to -0.44). It may also reduce the hazard of a renal composite endpoint by 16% (HR = 0.84, 95% CI 0.79 to 0.90) [15]. Although there were differences in the study populations of interest and statistical methods used, with similar sample sizes, our findings are consistent with the studies mentioned above.

The multivariable-adjusted Cox proportional hazards models and linear regression models showed a negative association between ALB and renal prognosis (HR = 0.61, 95%CI: 0.45–0.81, *P* < 0.001) and renal function decline (β = -1.41, 95%CI: -2.11 to -0.72, *P* < 0.001) in the present study after adjusting hypertension, age, diabetes, Hb, gender, BMI, use of CCB, history of CVD, UPCR, SBP, urinary occult blood, eGFR, use of RAAS inhibitor, and use of diuretics. Compared with those research, our study considered the effect of Hb, urinary occult blood, and baseline eGFR on the association between ALB and CKD progression when adjusting covariates. However, previous research considered these variables to be risk factors for CKD progression [43–45].

In addition, we explored the relationship between serum albumin and CKD progression in patients with varying stages of CKD or different UPCR levels through subgroup analysis. The interaction of ALB between the various CKD stages and UPCR groups was not statistically significant (*P* interaction > 0.05). Our findings extend the existing literature that supports the hypothesis that decreased serum albumin increases the hazard of CKD progression regardless of whether participants had different levels of baseline eGFR or proteinuria. The findings offered a guideline for clinical ALB level intervention to lower the hazard of CKD development.

Hypoalbuminemia possibly reflects several conditions: liver disease, poor nutrition, severe proteinuria, and chronic inflammation. Kadono et al. reported a significantly increased hazard of inflammation in patients with lower serum albumin concentrations [46]. Therefore, the mechanism of hypoalbuminemia on CKD progression may be related to the manifestations as mentioned above. Investigation in the Cardiovascular Health Study found that serum albumin was independently associated with a higher hazard of decreased renal function, while several markers of inflammation were not significantly associated [47]. However, the study did not simultaneously measure urinary albumin concentrations and thus could not discern the contribution of urinary albumin loss—an established prognostic marker and potential confounder of kidney disease. Unlike their study, we found an independent effect of serum albumin on renal prognosis and renal function decline in controlling proteinuria. Although the direct mechanism of action of hypoalbuminemia on CKD progression is unclear, it is clear that a rapid decline in renal function is closely associated with adverse renal outcomes [48]. The present study also found that patients who progressed to the renal composite endpoint experienced a faster decline in annual eGFR (Fig. S4). Combined with the results of this study, it is feasible that hypoalbuminemia could lead to adverse renal outcomes by accelerating the rate of eGFR decline in CKD patients.

Our study was the first to observe that serum albumin has a non-linear relationship with renal prognosis and renal function decline. The present study clarified a non-linear relationship between ALB and CKD progression. Our results showed that the serum albumin levels with the lowest log relative hazards and the annual rate of decline in eGFR was 4.1–4.3 g/dL. The reason for the difference in the relationship between serum albumin and CKD progression on either side of the inflection point is that other variables at the patient’s baseline may also influence the progression of CKD. It could be seen from Table S2 that compared with the ALB ≥ 4.3 g/dL, patients with ALB < 4.3 g/dL have generally higher age, UPCR, Scr levels, higher rates of diabetes, hypertension, diabetic nephropathy, history of CVD, urinary occult blood and use of diuretics. In contrast, patients generally had lower Hb and eGFR levels in the ALB < 4.3 g/dL group. However, the abnormality of the above indicators was closely associated with CKD progression [38, 39, 42, 44, 49–52]. When ALB was less than 4.3 g/dL, due to the presence of these CKD progression risk factors, increased hazard of CKD progression, and ALB as a protective factor for CKD progression, its negative association with CKD progression was relatively enhanced. The levels of risk factors for CKD progression were low when ALB was greater than 4.3 g/dL, including age, Scr, UPCR, diabetes, and hypertension. During this time, the protective effect of ALB was also weakened.

Our findings provide a rationale for slowing CKD progression by reducing the decline in ALB levels in the clinic, especially when ALB is below 4.1 g/dL. When ALB is lower than 4.1 g/dL, the hazard of CKD progression is at a high level. The inflection point provides evidence for serum albumin management. It offered more information on preventing CKD development in patients with varying serum albumin levels.

Our study has some strengths, which we have outlined below. (1) Most covariates have complete information, with only a few exceptions. To deal with missing data, multiple imputations were used. This method could maximize statistical power while minimizing potential bias caused by missing covariate information. (2) A series of sensitivity analyses were performed to assess the study’s robustness (target independent variable transformation, subgroup analysis, using a GAM to insert the continuity covariate into the equation as a curve, calculating E-values to investigate the possibility of unmeasured confounding, and reanalyzing the association between ALB and CKD progression after excluding participants with diabetic nephropathy or eGFR < 15 ml/min per 1.73 m^2^). (3) We analyzed the non-linear relationship in detail and identified the inflection point. (4) We carried out competing risks multivariate Cox’s regression with death as the competing hazard for the renal composite endpoint. (5) We simultaneously analyzed the relationship between serum albumin and renal prognosis and renal function decline to comprehensively explore the impact of serum albumin on the progression of CKD.

The following limitations of our study require addressed. First, the data was obtained from the study of CKD-ROUTE in Japan [17]. Therefore, we cannot conclude whether our findings apply to patients of different ethnicities and other regions. In addition, some patients withdraw their informed consent. The withdrawal reason might associate with the renal outcome. Second, because this was a secondary data analysis, variables not included in the original study, such as inflammatory factors, blood lipid levels, and diet status, could not be adjusted. We calculated E-value to assess the potential impact of unmeasured confounders and discovered that unmeasured confounders were unlikely to explain the results. Third, serum albumin level could be influenced by different factors on its synthesis, break down, excretion as proteinuria, etc. Under various nutritional disorders, inflammatory conditions, and most critical glomerular diseases of proteinuria, and under such circumstances, using extensive adjustment of covariates in statistical analyses still cannot address the role of albumin level in relation to disease outcomes from pathogenesis point of view or from mechanisms of disease progress, especially the renal outcomes. Fourth, the diagnosis of the attending physician identified the etiology of CKD. Many patients do not have renal biopsies. Fifth, patients with CKD stages 2–5 were included in the current study. All patients had an eGFR of less than 90. The relationship between ALB and CKD progression in patients with CKD stage 1 still needs to be studied further. Sixth, in the present study, 372 patients were excluded because of a loss of follow-up. This might lead to lost follow-up bias. When we compared patients who stayed in the final analysis with patients who were lost to follow-up, we found that patients who lost to follow-up were older and had lower hemoglobin and ALB levels. These indicators are all associated with CKD progression. In fact, the proportion of these patients progressing to the renal composite endpoint was lower. Therefore, we are inclined to think that the bias caused by lost follow-up is likely to be small. Seventh, the current study only measured serum albumin at the baseline and did not consider changes in ALB over time. In the future, we can design our investigations to incorporate indicators such as inflammatory and nutritional factors and focus on the impact of dynamic changes in ALB on CKD progression.

## Conclusion

This study demonstrates a negative and non-linear relationship between serum albumin and renal prognosis and renal function decline in the Japanese population. There is a saturation effect between serum albumin levels and CKD progression. When serum albumin level is less than 4.1 g/dL, a decrease in serum albumin is closely associated with poor renal prognosis and an increased rate of renal function decline. This result is expected to provide a reference for clinicians to intervene in serum albumin levels. From a treatment perspective, it makes sense to avoid serum albumin levels below the inflection point.

## Supplementary Information

**Additional file 1: Table S1.** Baseline characteristics of the patients with or without lost-follow up. **Table S2.** The Baseline Characteristics of participants on both sides of the inflection point. **Figure S1.** Distribution of serum albumin in different renal prognosis. **Figure S2.** Renal composite endpoint incidence rate of age stratification by 20 intervals. **Figure S3.** Average annual eGFR decline of age stratification by 20 intervals. **Figure S4.** The mean annual eGFR decline according to progression to the renal composite endpoint or not.

## Data Availability

The data used in the study is from a publicly available database, which can be downloaded from https://datadryad.org/stash/dataset/doi:10.5061%2Fdryad.kq23s.

## Abbreviations

Hb: Hemoglobin
UACR: Urine albumin creatine ratio
UPCR: Urinary protein-to-creatinine ratio
CKD: Chronic kidney disease
KDIGO: Kidney Disease Improving Global Outcomes
CKD-ROUTE: Chronic kidney disease research of outcomes in treatment and epidemiology
BMI: Body mass index
DKD: Diabetic kidney disease
ESRD: End-stage renal disease
GAM: Generalized additive model
Scr: Serum creatinine
SBP: Systolic blood pressure
eGFR: Estimated glomerular filtration rate
DBP: Diastolic blood pressure
ALB: Serum albumin
ARB: Angiotensin receptor blockers
CCB: Calcium channel blocker
MI: Myocardial infarction
ACEI: Angiotensin-converting enzyme inhibitors
CVD: Cardiovascular disease
CHF: Congestive heart failure
HR: Hazard ratios
PAD: Peripheral arterial disease
AP: Angina pectoris
CI: Confidence intervals
SHR: Subdistribution hazard ratios
