# Supplementary material for: The level of serum albumin is associated with renal prognosis and renal function decline in patients with chronic kidney disease
Source: BMC Nephrol. 2023 Mar 15;24:57. doi: 10.1186/s12882-023-03110-8 (PMC10018824; doi:10.1186/s12882-023-03110-8)
Supplement: Supplementary file 1 — Additional file 1: Table S1. Baseline characteristics of the patients with or without lost-follow up. Table S2. The Baseline Characteristics of participants on both sides of the inflection point. Figure S1. Distribution of serum albumin in different renal prognosis. Figure S2. Renal composite endpoint incidence rate of age stratification by 20 intervals. Figure S3. Average annual eGFR decline of age stratification by 20 intervals. Figure S4. The mean annual eGFR decline according to progression to the renal composite endpoint or not. [file 12882_2023_3110_MOESM1_ESM.docx]

**The level of serum albumin is associated with renal prognosis and renal function decline in patients with chronic kidney disease**

**Running title:** The relationship between serum albumin and CKD progression

**Tong Cheng^1^**^#^**, Xiaoyu Wang^2#^, Yong Han^3^**^#^**, Jianbing Hao^1^, Haofei Hu^4^*, Lirong Hao^1^***

^1^Department of Nephrology, Southern University of Science and Technology Hospital, Shenzhen, 518000, Guangdong Province, China

^2^Department of Nephrology, Hechi People's Hospital, Hechi 547000, Guangxi Zhuang Autonomous Region, China

^3^Department of Emergency, Shenzhen Second People’s Hospital, Shenzhen 518000, Guangdong Province, China

^4^Department of Nephrology, Shenzhen Second People’s Hospital, Shenzhen 518000, Guangdong Province, China

**^#^** Tong Cheng, Xiaoyu Wang and Yong Han have contributed equally to this work.

***Corresponding author**

Haofei Hu,

Department of Nephrology,

Shenzhen Second People’s Hospital,

No.3002 Sungang Road, Futian District,

Shenzhen 518000,

Guangdong Province,

China

Tel:+86-755-83366388

E-mail: huhaofei0319@126.com

**Lirong Hao,**

Department of Nephrology,

Southern University of Science and Technology Hospital,

No.6019 Liuxian Street, Xili Avenue, Nanshan District,

Shenzhen 518000,

Guangdong Province,

China

E-mail: hao_lirong@163.com

**Table S1. Baseline characteristics of the patients with or without lost-follow up**

| Variables | Not lost to follow-up | Lost to follow up | P-value |
| --- | --- | --- | --- |
| Age(years) | 66.90 ± 13.41 | 68.97 ± 13.95 | <0.05 |
| SBP(mmHg) | 139.83 ± 21.88 | 139.93 ± 23.78 | 0.94 |
| BMI(kg/m^2^) | 23.89 ± 3.80 | 23.41 ± 4.50 | 0.08 |
| HB(g/dL) | 12.14 ± 2.17 | 11.53 ± 2.47 | <0.001 |
| ALB(g/dL) | 3.89 ± 0.59 | 3.76 ± 0.72 | <0.05 |
| eGFR (ml/min per 1.73 m2) | 33.35 ± 17.97 | 31.42 ± 20.08 | 0.11 |
| UPCR (g/gCr) | 0.74 (0.14-2.62) | 0.73 (0.10-3.48) | 0.70 |
| Gender |  |  | 0.75 |
| Male | 522 (69.14%) | 260 (70.08%) |  |
| Female | 233 (30.86%) | 111 (29.92%) |  |
| Etiology of CKD |  |  | 0.46 |
| Diabetic nephropathy, n(%) | 183 (24.24%) | 104 (28.03%) |  |
| Nephrosclerosis, n (%) | 305 (40.40%) | 140 (37.74%) |  |
| Glomerulonephritis, n (%) | 148 (19.60%) | 65 (17.52%) |  |
| Other, n (%) | 119 (15.76%) | 62 (16.71%) |  |
| Urinary occult blood, n(%) | 239 (31.95%) | 138 (37.60%) | 0.06 |
| Hypertension, n (%) | 684 (90.60%) | 333 (89.76%) | 0.65 |
| History of CVD, n (%) | 194 (25.70%) | 107 (28.84%) | 0.26 |
| Diabetes, n (%) | 278 (36.82%) | 142 (38.27%) | 0.64 |
| Renal composite endpoint, n (%) | 220 (29.14%) | 58 (15.63%) | <0.001 |

Continuous variables are presented as mean ± standard deviation and median with interquartile ranges. Categorical data are presented as numbers and percentages.

Abbreviations: SBP, Systolic blood pressure; BMI, body mass index; HB, Hemoglobin; ALB, Serum albumin; CKD, chronic kidney disease; eGFR, estimated glomerular filtration rate; CVD, cardiovascular disease; UPCR, urinary protein/creatinine ratio; g/gCr, gram per gram creatinine;

## Table S2 The Baseline Characteristics of participants on both sides of the inflection point.

| ALB(g/dL) | <4.3 | >=4.3 | P-value |
| --- | --- | --- | --- |
| Participants | 528 | 226 |  |
| Age(years) | 68.11 ± 12.85 | 63.93 ± 14.23 | <0.001 |
| SBP(mmHg) | 140.74 ± 22.36 | 137.68 ± 21.23 | 0.08 |
| BMI(kg/m^2^) | 23.79 ± 3.81 | 23.70 ± 4.00 | 0.78 |
| HB(g/dL) | 11.71 ± 2.12 | 13.18 ± 1.97 | <0.001 |
| ALB(g/dL) | 3.63 ± 0.51 | 4.48 ± 0.19 | <0.001 |
| Scr(mg/dL) | 1.88 (1.30-2.84) | 1.30 (1.07-1.89) | <0.001 |
| eGFR (ml/min per 1.73 m2) | 30.28 ± 16.86 | 40.77 ± 18.37 | <0.001 |
| UPCR (g/gCr) | 1.26 (0.30-3.95) | 0.20 (0.05-0.72) | <0.001 |
| Gender |  |  | 0.94 |
| Male | 366 (69.32%) | 156 (69.03%) |  |
| Female | 162 (30.68%) | 70 (30.97%) |  |
| Etiology of CKD |  |  | <0.001 |
| Diabetic nephropathy, n(%) | 162 (30.68%) | 20 (8.85%) |  |
| Nephrosclerosis, n (%) | 192 (36.36%) | 113 (50.00%) |  |
| Glomerulonephritis, n (%) | 103 (19.51%) | 44 (19.47%) |  |
| Other, n (%) | 71 (13.45%) | 49 (21.68%) |  |
| Urinary occult blood, n(%) | 194 (36.74%) | 49 (21.68%) | <0.001 |
| Hypertension, n (%) | 491 (92.99%) | 192 (84.96%) | <0.001 |
| History of CVD, n (%) | 152 (28.79%) | 41 (18.14%) | <0.05 |
| Diabetes, n (%) | 222 (42.05%) | 55 (24.34%) | <0.001 |
| Use of RAAS inhibitor, n(%) | 367 (69.51%) | 137 (60.62%) | <0.05 |
| Use of calcium channel blocker, n (%) | 283 (53.60%) | 98 (43.36%) | <0.05 |
| Use of diuretics, n (%) | 187 (35.42%) | 52 (23.01%) | <0.001 |
| CKD stage, n (%) |  |  | <0.001 |
| 2 | 31 (5.87%) | 31 (13.72%) |  |
| 3 | 205 (38.83%) | 122 (53.98%) |  |
| 4 | 189 (35.80%) | 61 (26.99%) |  |
| 5 | 103 (19.51%) | 12 (5.31%) |  |

Continuous variables are presented as mean ± standard deviation and median with interquartile ranges. Categorical data are presented as numbers and percentages.

Abbreviations: BMI, body mass index; SBP, Systolic blood pressure; Scr, Serum creatinine; ALB, Serum albumin; HB, Hemoglobin; CKD, chronic kidney disease; CVD, cardiovascular disease; eGFR, estimated glomerular filtration rate; UPCR, urinary protein/creatinine ratio; g/gCr, gram per gram creatinine; RAAS, renin-angiotensin aldosterone system.

**
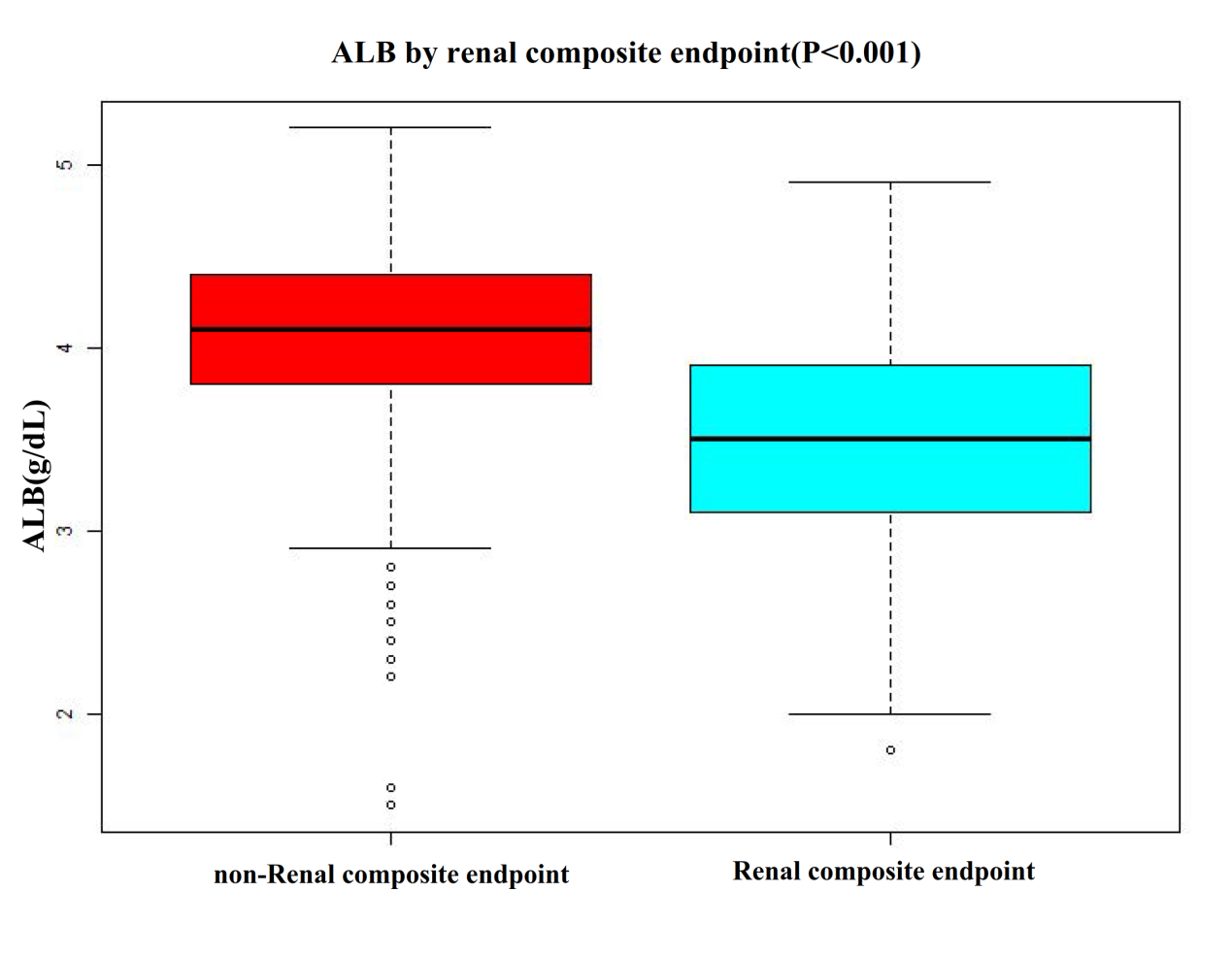
**

**Figure S1. Distribution of serum albumin in different renal prognosis.**

**
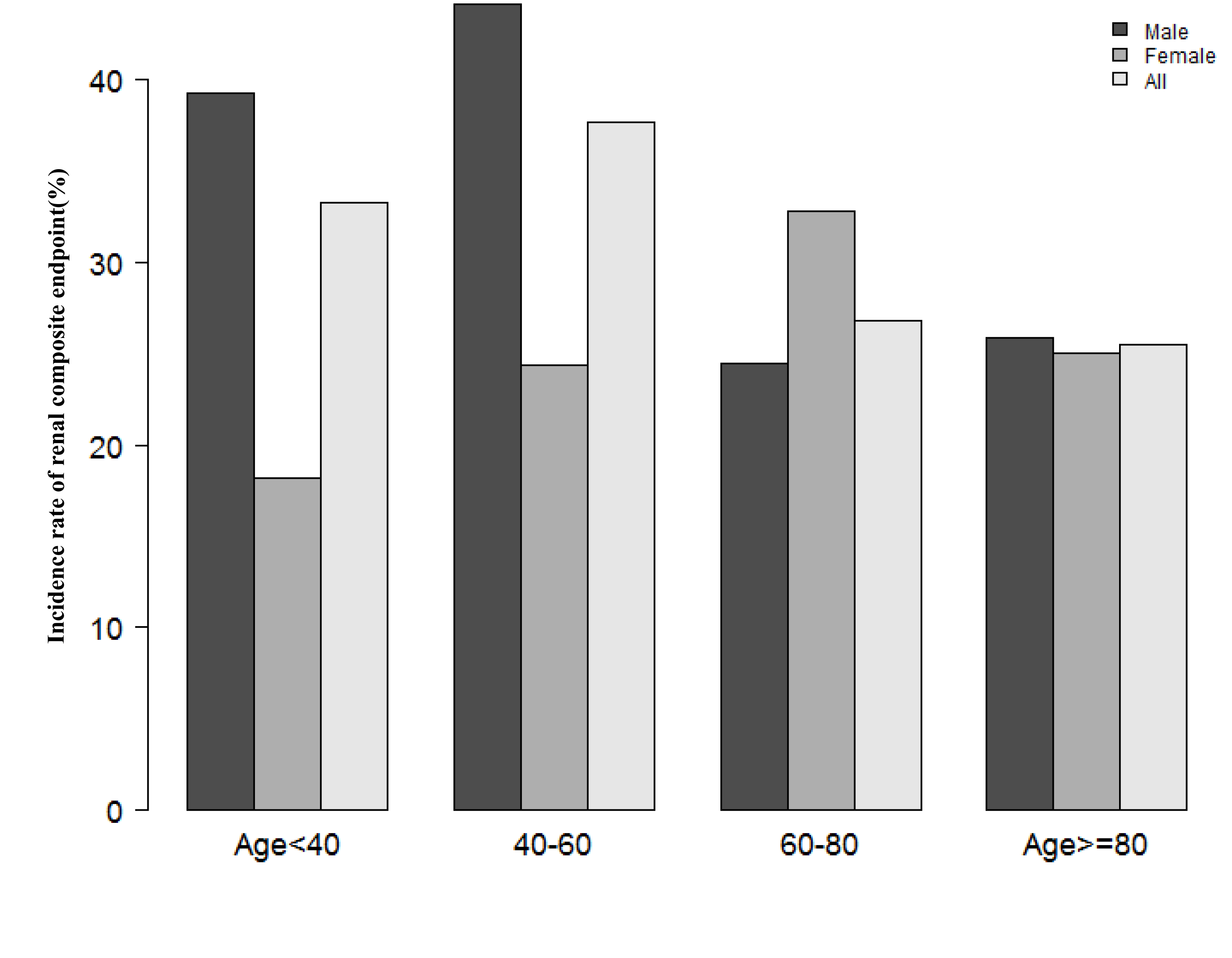
**

**Figure S2. Renal composite endpoint incidence rate of age stratification by 20 intervals.**

**
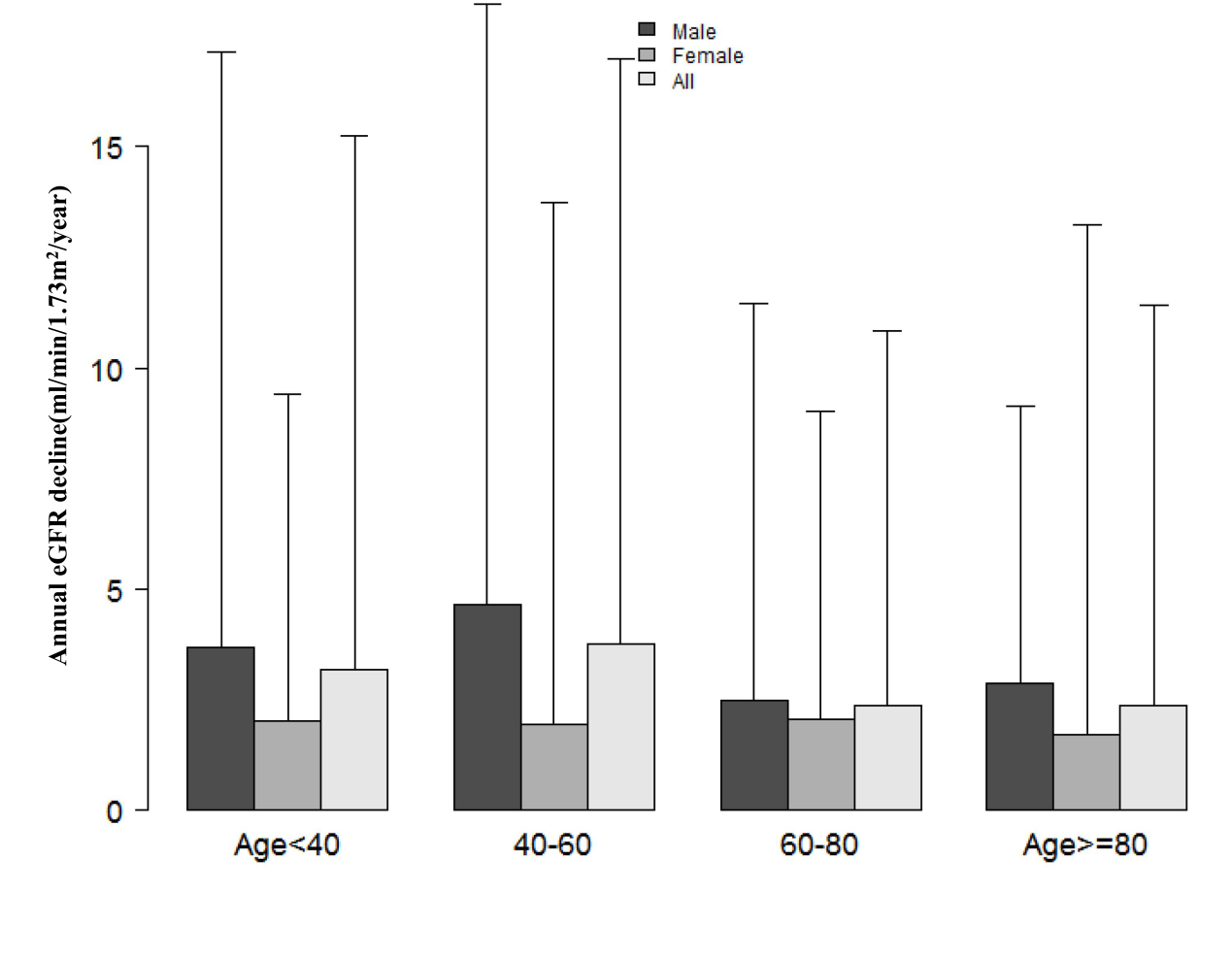
**

**Figure S3. Average annual eGFR decline of age stratification by 20 intervals.**

**
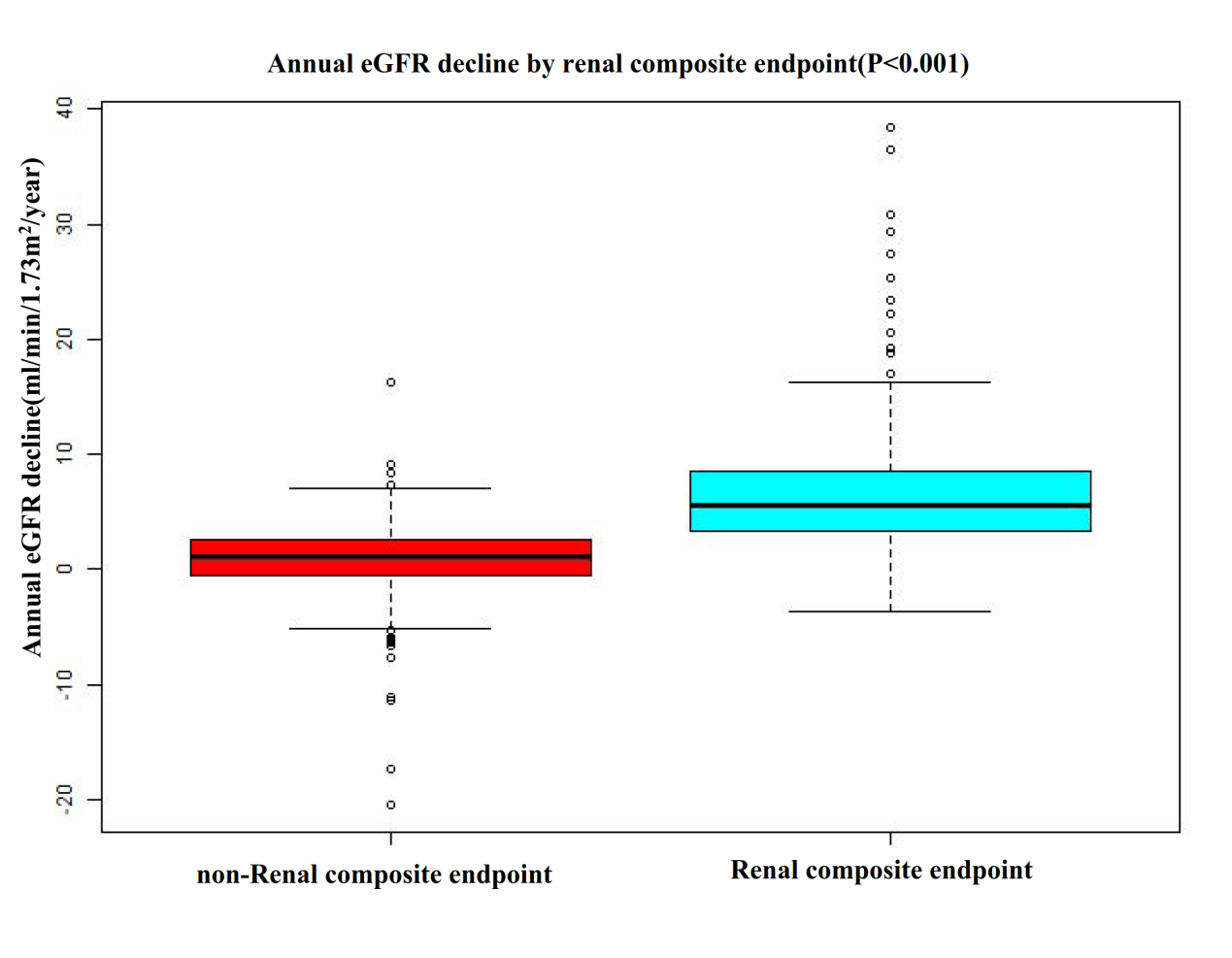
**

**Figure S4.** **The mean annual eGFR decline according to progression to the renal composite endpoint or not.**
